# The impact of organizational politics on work engagement—the mediating role of the doctrine of the mean

**DOI:** 10.3389/fpsyg.2023.1283855

**Published:** 2023-12-18

**Authors:** Weijian Su, Chengxuan Xie

**Affiliations:** ^1^Economic Research Center, Shandong University of Finance and Economics, Jinan, China; ^2^Veterans Education College, Tangshan Open University, Tangshan, Hebei, China

**Keywords:** organizational politics, work engagement, the doctrine of the mean, cultural differences, library personnel

## Abstract

**Introduction:**

Events Theory posits that the perception of organizational politics affects job attitudes. The purpose of this study was to answer the question: does organizational politics have a similar impact on Chinese individuals who adhere to the doctrine of the mean?

**Methods:**

We collected survey data from 3,059 library personnel in 36 Chinese university libraries and conducted analysis using the Analytic Hierarchy Process.

**Results:**

The results showed that the perception of organizational politics reduces employee work engagement. However, this impact exhibits heterogeneity. For newly recruited employees and employees aged 50 and above who are nearing retirement, the impact of organizational politics on work engagement is relatively small. Conversely, for employees aged 30–50, organizational politics has a significant negative impact. Furthermore, the doctrine of the mean moderates the impact of organizational politics on work engagement, mitigating its negative effects.

**Discussion:**

The conclusions offer new insights into enhancing employee work motivation. The library should take more measures to safeguard employee rights to enhance work motivation.

## 1 Introduction

The growth and flourishing of an organization significantly depend on employee work engagement (Atshan et al., 2022). While various factors such as the work environment, salary levels, and task complexity can influence employee enthusiasm, the importance of organizational politics is increasingly garnering scholarly attention (Ellen et al., 2022; Ali et al., 2023). This is primarily because organizational politics has the potential to impact employee performance, attitudes, efforts, and loyalty (Bhattarai, 2023). From a theoretical perspective, the Person-Organization Fit theory posits that when an individual’s values, work attitudes, and career goals align with the culture, values, and objectives of their respective organization, it is likely to result in higher job satisfaction, lower turnover rates, and improved job performance (Kristof-Brown et al., 2023; Manzi et al., 2023). Therefore, investigating the impact of organizational politics on employee work engagement becomes a highly meaningful research topic (Guo et al., 2019).

While numerous studies have analyzed the impact of organizational politics on job engagement (Alsarhan and Valax, 2021; Khan et al., 2021), the findings are not consistent. Eldor (2017), Kundi et al. (2023) both found that the perception of organizational politics enhances employee engagement and knowledge sharing. However, most studies, such as Khan et al. (2018), and Dappa et al. (2019) indicate that organizational politics tends to decrease employee job engagement.

Mehmood et al. (2023) provided an explanation for the contradictory conclusions. They suggested that when employees perceive the existence of organizational politics, their varied responses might lead to differential effects on job engagement. For instance, when employees adapt positively to the organizational political culture and use less legitimate or covert means to achieve personal work goals, organizational politics may enhance employee motivation (Mehmood et al., 2023). On the other hand, when employees adopt a more negative and confrontational attitude, or even plan to leave the organization, organizational politics could reduce job engagement (Pinnington et al., 2023). In other words, prior research may not have adequately incorporated employees’ attitudes toward organizational politics into their study frameworks, which resulted in less persuasive conclusions. Furthermore, Ahmad et al. (2023) emphasizes that employees’ attitudes toward organizational politics are closely linked to their personal values and cultural backgrounds. For instance, some employees may strongly disapprove of practices such as bribery, deception, or unethical competition, while others may perceive these behaviors as normal (Scharding and Warren, 2023; Tan et al., 2023). If employees strongly believe in fairness and justice, they are likely to hold a negative view of organizational politics, reflecting the influence of their personal values (Kaur and Kang, 2023).

It is well known that an individual’s attitude and judgment toward things are related to their cultural background (Gregg et al., 2022). In other words, the effect of organizational politics on work engagement may be influenced by cultural context. However, when examining the influence of cultural background differences, research on how organizational politics affects job engagement is relatively scarce. But Khan et al. (2018, 2021), Irangani et al. (2021), De Clercq et al. (2023), and Bhattarai (2023) made pioneering contributions in this aspect. For the first time, Khan et al. (2018) integrates psychological contract breach, political skill, and work ethic into the framework, examining how these factors play a role in the impact of organizational politics on employee job attitudes, work ethic is, in fact, a set of values and a reflection of cultural background, because work ethics vary across different cultural backgrounds. Furthermore, Khan et al. (2021) discovered that work ethic weakens the relationship between perceived organizational politics and job satisfaction, analyzing a sample of 310 faculty members from higher education institutions in Pakistan. Irangani et al. (2021) analyzed the impact of the competitive psychological climate on perceived effects, suggesting that an individual’s level of perception of organizational politics may be linked to their psychological state. De Clercq et al. (2023) analyzed the role of tenacity and passion for work in how organizational politics affect employee performance, while Bhattarai (2023) examined the impact of self-promotion behavior. These studies imply that in researching how organizational politics affect work engagement, it is necessary to consider the impact of cultural differences. In fact, the phenomenon of organizational politics is more prevalent in East Asian countries, particularly in China, South Korea, and Japan (Kim et al., 2016; Hemmert et al., 2019). In China, in particular, the Confucian value system has a history of over five thousand years, providing a good case for analyzing the potential impact of organizational politics on job engagement in different cultural settings. Thus, the central focus of this study is to investigate whether the doctrine of the mean, which is the core philosophy of Confucianism value system, have an impact on how organizational politics affects job engagement. In this study, we selected library staff members at Chinese universities as our research subjects. This choice was primarily due to their status as part of the public sector workforce and the monotonous nature of their job roles. And improving their work efficiency is an important issue.

This research makes three marginal contributions. First, this study extends the research of Khan et al. (2021), expanding the focus from work ethic to the doctrine of the mean. Second, it complements the findings of Romani et al. (2018) and Hsiao (2022) by enriching the study of the impact of organizational politics across cultural backgrounds. Third, we discover that the doctrine of the mean moderates the negative effects of organizational politics, highlighting the importance of cultural background in moderating the influence of organizational politics and providing new evidence to support the shaping of organizational culture and cross-cultural management.

## 2 Literature review and hypotheses development

### 2.1 Impact of organizational politicson work engagement

Job involvement refers to the degree of an individual’s psychological and emotional engagement with their work (Kossyva et al., 2023). High job involvement can enhance productivity, reduce turnover rates, and increase employee job satisfaction (Bhakuni and Saxena, 2023; Yunike et al., 2023). Consequently, this leads to a reduction in occupational burnout and an increase in job-related well-being. The Person-Organization Fit Theory suggests that the alignment between individual and organizational values, goals, attitudes, and enthusiasm is a critical determinant of employee attitudes and behaviors (Kim and Park, 2017). High alignment occurs when individual values, goals, attitudes, and work enthusiasm align with the organization’s culture, atmosphere, objectives, and norms, or when the needs of either the individual or the organization are met by the other party (Poliakova, 2022; Tourky et al., 2023). Such alignment results in higher situational adaptability, leading to positive impacts on work attitudes and behaviors (Mendiratta and Srivastava, 2023).

However, when organizational political behavior is prevalent within the organization, where employees frequently resort to covert and non-traditional means to achieve personal goals, such as job transfers or promotions, this type of organizational political behavior can have detrimental effects on other employees (Ahmad et al., 2023). It disrupts their established psychological perceptions of fairness and justice, potentially eroding their work enthusiasm and overall positivity (Alamoush et al., 2021).

Despite some scholars, such as Oubibi et al. (2022), Munoz et al. (2022), emphasizing that certain employees may actively adapt and engage in organizational political behavior to achieve personal goals and subsequently increase work engagement. Tewolde (2023) argues that in most countries, the majority of residents oppose and detest the phenomenon of organizational politics. Furthermore, Lalive et al. (2023) observes that employees nearing retirement may exhibit indifference to organizational politics, implying that those still actively employed might be more sensitive to such dynamics. Based on the above analysis, we propose the first research hypothesis:

**H1:** Organizational politics reduces employee work engagement.

**H2:** The impact of organizational politics on work engagement may vary due to differences in age.

### 2.2 The influence of the doctrine of the mean

The core principle of the doctrine of the mean is to avoid extremes, adapt to the environment, and maintain harmony without force (Li and Cui, 2022). It shapes the Chinese perception and attitude toward things and interactions, including views on job positions and attitudes toward work engagement (Ho and Barton, 2022). The concept of the doctrine of the mean holds significant relevance in understanding individual behaviors and attitudes within organizations, particularly in China (Gao et al., 2022).

Niemiec (2019) and Zhang et al. (2021) found that if employees adhere to the doctrine of the mean, even when they perceive the presence of organizational politics, it does not have a negative impact on their work engagement. This is because the doctrine of the mean allows them to calmly assess personal gains and losses and cope with injustices through self-psychological comfort. In other words, the doctrine of the mean mitigates the negative effects of organizational politics on employee work engagement (Khan et al., 2021).

Another noteworthy phenomenon is the gender difference in the influence of the doctrine of the mean. In China, women are characterized by qualities, such as diligence, courage, and a willingness to take responsibility (Basu et al., 2017; Qian and Florence, 2021). Abundant social observations suggest that, compared to men, women tend to be more rational in problem-solving (Zhang et al., 2019; Yoo, 2020), making their behavior more aligned with the doctrine of the mean. Based on the above analysis, we propose the following hypothesis:

**H3:** The doctrine of the mean can mitigate the impact of organizational politics on employee work engagement.

**H4:** The moderating effect of the doctrine of the mean is subject to gender differences.

## 3 Research design

### 3.1 Sample and data

In this study, 36 university and college libraries in China were selected as our research sample, these universities are located in Shandong Province, Hebei Province. Primary data were collected by a self-administered questionnaire in year 2022, resulting in a collection of 3,127 responses. Similar to the studies conducted by Khan et al. (2021), purposive sampling technique was employed.

To reduce common method bias, measures such as ensuring anonymity and privacy protection and controlling relevant variables are implemented. Given the sensitive nature of organizational politics, the questionnaires were filled out anonymously to ensure the authenticity of the responses. The participants were assured that the collected data would be used exclusively for academic research and that the privacy of their responses would be strictly protected.

To ensure the validity of our sample selection, we rigorously filtered the data according to exclusion criteria suggested by Ferris et al. (2019): (1) Data with basic information errors were discarded. For example, if a university was located in Qingdao but a respondent incorrectly marked it as Jinan, such responses were considered unreliable and hence, 7 questionnaires were excluded. (2) Questionnaires with identical answers throughout were eliminated, as this indicated respondents might have answered arbitrarily without genuinely expressing their views, leading to the removal of 32 questionnaires. (3) Partially answered questionnaires were also discarded, as they could cause regression bias and affect the reliability of the results, resulting in 29 questionnaires being removed. Consequently, a total of 3,059 valid samples were obtained. Currently, the use of survey data to study issues of organizational politics is a prevalent approach in academia, as demonstrated in the research of Malik et al. (2019) and Hochwarter et al. (2020). The overall response rate was 97% (*N* = 3127).

### 3.2 Definition of variables

#### 3.2.1 Work engagement (WE)

The study employed the scale developed by Schaufeli et al. (2002), comprising 24 items to assess employee job involvement and fatigue. These items are designed to reflect three latent dimensions: Vigor (VI) with 9 items (e.g., “When I wake up in the morning, I feel like going to work”), Dedication (DE) with 8 items (e.g., “I am enthusiastic about my library management job”), and Absorption (AB) with 7 items (e.g., “I get lost in my work, forgetting about everything else”). The scoring method for these engagement items is aligned with that of the MBI-GS. The Cronbach’s α of this scale is 0.81.

#### 3.2.2 Organizational politics (OP)

The Organizational Politics variable was measured using the scale developed by Kacmar and Carlson (1997), consisting of 14 items. These items encompass three dimensions: General Political Behavior Perception (2 items), Perception of Silent Waiting for Opportunities (7 items), and Perception of Political Payoffs and Promotion Policies (5 items). Notably, the first two items of the latter two dimensions are reverse coded, requiring reverse scoring for accurate assessment. The Cronbach’s α of this scale is 0.91.

#### 3.2.3 The doctrine of the mean (MGL)

In this study, the “Moderation Relationship Scale” developed by Buskell (2019) was employed. Comprising 13 items, the scale includes queries such as “Is it more reasonable or appropriate to consider working harmoniously with colleagues, or are both aspects equally important?”, “Is it necessary to consider colleagues’ feelings before deciding how to handle a task?”, and “How do you persuade others to accept your suggestions when they disagree with your opinions or methods?” Following the approach of Basu et al. (2017), respondents rated their agreement with each statement on a seven-point scale, with higher values indicating stronger support for the description. This scale has been commended by Dodier et al. (2022) for its reliability and widely recognized in academic research.

In the present study, we utilized the “Moderation Relationship Scale” developed by Buskell (2019), which consists of 13 items. This scale poses questions such as “Is it more reasonable or appropriate to work harmoniously with colleagues”, or “is it equally important to consider other aspects?”, “How important is it to take into account colleagues feelings before deciding on a course of action?”, and “What strategies do you employ to convince others when they disagree with your views or methods?”. In alignment with the methodology proposed by Tewolde (2023), participants expressed their level of agreement with each item using a seven-point Likert scale, where higher scores denote greater endorsement of the statements. The scale has been lauded for its reliability by Dodier et al. (2022) and is well-regarded in the field of academic research.

#### 3.2.4 Control variables

In terms of control variable selection, to minimize the influence of individual employee characteristics on work engagement, the questionnaire surveyed respondents’ age, gender, and education level. The variable AGE represents the employee’s age. GENDER is a dummy variable, where 0 indicates male and 1 indicates female. EDUCATION denotes the level of education, with 0 for sub-diploma, 1 for diploma, 2 for bachelor’s degree, and 3 for postgraduate degree.

#### 3.2.5 Demographic information

In the sample, there were 22 employees with postgraduate degrees, accounting for 0.7%; 2,376 employees held bachelor’s degrees, making up 77.7%. Female employees numbered 1,072, representing 18.17%.

## 4 Results

### 4.1 Data quality analysis

#### 4.1.1 Confirmatory factor analysis

The initial step involved a confirmatory factor analysis, the results of which are displayed in Table 1. It was observed that, except for the factor loadings of VI3, OP3, and DM5, which fell below 0.6, all other factor loadings were above 0.6, with the majority exceeding 0.7. This exceeds the threshold of 0.3 for factor loadings suggested by Hair and Sarstedt (2014), confirming the validity of the latent variable constructs.

**TABLE 1:** Convergent validity: factor loadings, average variance extracted (AVE), and construct reliability of scale.

Variables	Items	Factor loading	AVE score	CR values
WE			0.61	0.90
	VI1	0.72		
	VI2	0.68		
	VI3	0.57		
	VI4	0.77		
	VI5	0.90		
	VI6	0.71		
	VI7	0.76		
	VI8	0.73		
	VI9	0.69		
	DE1	0.65		
	DE2	0.59		
	DE3	0.75		
	DE4	0.84		
	DE5	0.78		
	DE6	0.77		
	DE7	0.83		
	DE8	0.76		
	AB1	0.86		
	AB2	0.86		
	AB3	0.73		
	AB4	0.69		
	AB5	0.74		
	AB6	0.75		
	AB7	0.81		
OP			0.87	0.95
	OP1	0.89		
	OP2	0.75		
	OP3	0.58		
	OP4	0.66		
	OP5	0.79		
	OP6	0.87		
	OP7	0.88		
	OP8	0.85		
	OP9	0.87		
	OP10	0.88		
	OP11	0.84		
	OP12	0.91		
	OP13	0.82		
	OP14	0.81		
MGL			0.79	0.92
	MGL1	0.87		
	MGL2	0.81		
	MGL3	0.78		
	MGL4	0.73		
	MGL5	0.57		
	MGL6	0.69		
	MGL7	0.85		
	MGL8	0.86		
	MGL9	0.79		
	MGL10	0.87		
	MGL11	0.91		
	MGL12	0.68		
	MGL13	0.70		

The Average Variance Extracted (AVE) values exceeded 0.60, surpassing the accepted threshold of 0.5, indicating high convergent validity of the scales. Composite Reliability (CR) values were above 0.90, well over the standard criterion of 0.7, demonstrating high reliability of the variables. These findings justify further analysis using the data. It is noteworthy that the selection of minimum thresholds for factor loadings, AVE, and CR values in this study aligns with the criteria set by Mahembe and Engelbrecht (2013).

#### 4.1.2 Descriptive statistics and Pearson correlation coefficients

Table 2 presents the descriptive statistics and Pearson correlation coefficients for the variables. The correlation matrix reveals a negative correlation between Work Engagement (WE) and Organizational Politics (OP), significant at the 5% level. This finding preliminarily suggests that organizational politics may negatively impact employee engagement, warranting further investigation. Additionally, a positive correlation is observed between the doctrine of the mean and Work Engagement, and a negative correlation with Organizational Politics. These results indicate a potential moderating effect of the doctrine of the mean.

**TABLE 2:** Descriptive statistics and Pearson correlation coefficients.

	Mean	S.D.	1	2	3	4	5	6
1. WE	-0.061	0.993	1					
2. OP	0.875	0.525	-0.473**	1				
3. MGL	0.026	1.080	0.321***	-0.273**	1			
4. AGE	37.690	10.157	0.167	0.496**	0.587	1		
5. GENDER	1.842	0.533**	-0.073	0.530	-0.212	0.765**	1	
6. EDUCATION	0.673	0.473	0.906	-0.051**	0.805	0.116	0.653*	1

*indicates *p* < 0.05,

**indicates *p* < 0.01,

***indicates *p* < 0.001.

We also assessed the potential issue of multicollinearity in the data by conducting a Variance Inflation Factor (VIF) analysis to enhance the reliability of our analytical results. The highest VIF value observed was 5.271, which falls below the commonly accepted threshold of 10. This indicates that multicollinearity is not a concern in our dataset.

### 4.2 Regression results

#### 4.2.1 Hierarchical regression

Hypothesis 1 suggests a negative influence of organizational politics on work engagement. The analysis in this study was conducted using Spss and Stata software. Table 3 presents the results. In column (1), the outcomes are displayed without any control variables. According to the prior definition of organizational politics, if it indeed has a negative impact on work engagement among library management personnel, its coefficient should be negative. As shown in column (1), the coefficient of OP is negative and significant at the 1% level. The right-hand side of column (1) displays the *T*-values corresponding to the coefficients of each variable, indicating their significance levels. Furthermore, in column (2), the results are reported after adding control variables such as AGE, EDUCATION, and GENDER. These results provide additional evidence to support the idea that organizational politics may reduce work engagement.

**TABLE 3:** Results of the influence of organizational politics on work engagement.

Variables	WE			
	(1)		(2)	
	Coeff	*T*-value	Coeff	*T*-value
OP	-0.370***	-2.63	-0.363**	-2.53
AGE			-0.005	-0.87
EDUCATION			-0.081**	-2.08
GENDER			0.050	0.41
Constant	-0.380**	-2.59	-0.058	-0.13
*N*	3059		3059	
R^2^	0.234		0.229	

**indicates *p* < 0.01,

***indicates *p* < 0.001.

#### 4.2.2 Heterogeneity analysis

As discussed earlier, newly recruited young library management personnel, fresh out of university and adapting to a new work environment, often display high levels of enthusiasm and motivation. To investigate whether the impact of organizational politics on work engagement varies based on age, we conducted subgroup analyses within the entire sample.

Specifically, employees were categorized into different age groups. Individuals below the age of 30 were classified as the “early-career” group, those aged between 30 and under 50 were identified as the “mid-career” group, and those aged 50 and above were categorized as the “approaching retirement” group. Notably, the 30–50 age range was chosen as the reference group for comparison. This decision was made because, compared to employees above 30, personnel under 30 typically have shorter job tenures and limited social experience.

Table 4, columns (1) and (2), along with column (3), present the effects of organizational politics on work engagement for the different age groups, respectively. In the regression for the group below 30, the coefficient of OP, while negative, is not significant, because the *T*-value is only −1.14. For the group aged 30 to under 50, the results align with the findings from the analysis of the full sample. Similarly, in the group aged 50 and above, the coefficient remains insignificant. These findings prove the predictions made in the theoretical analysis section. Young employees who are newly recruited exhibit high enthusiasm but have limited professional experience. Their sensitivity to organizational politics appears to be weaker than that of those aged above 30. This is because individuals aged 30 to under 50 are typically in a critical phase of career development or consolidation, making them more attuned to perceptions of organizational politics. If informal organizational behaviors exceed formal regulations, the negative impact on their work attitude and motivation becomes more apparent. As for the group aged 50 and above, even if they sense the presence of organizational politics, due to their impending retirement, they may remain relatively indifferent. This results in an insignificant influence of organizational politics on work engagement. The results mentioned above validate the accuracy of H1 and H2.

**TABLE 4:** Heterogeneity analysis results.

	(1)		(2)		(3)	
	Below 30		Aged 30–50		Aged 50 and above	
Variables	Coeff	*T*-value	Coeff	*T*-value	Coeff	*T*-value
OP	-0.298	-1.14	-0.413**	-2.27	-0.504	-1.52
AGE	-0.070	-1.40	-0.004**	-2.16	0.013	0.23
EDUCATION	-0.115	-0.15	-0.057**	-2.09	-0.210	-0.96
GENDER	-0.139	-0.54	0.128	0.92	-0.151	-0.61
Constant	1.837	0.97	-0.227	-0.35	-0.864	-0.28
*N*	1043		1144		872	
R^2^	0.203		0.239		0.273	

**indicates *p* < 0.01,

#### 4.2.3 The moderating effect of the doctrine of the mean

Hypothesis 3 posits that if personnel adopt the doctrine of the mean as a guiding principle in their interactions with others, the doctrine of the mean would weaken the negative impact of organizational politics on work engagement. In other words, the doctrine of the mean moderates the adverse effects of organizational politics on work engagement, suggesting the presence of a moderating effect.

**FIGURE 1:**
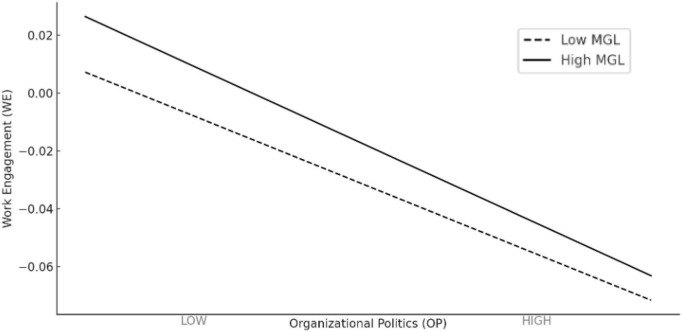
Moderating effect of MGL on the relationship between OP and WE.

To verify the validity of this conjecture, we augmented the original regression analysis by introducing the moderate variable and its interaction with the organizational politics variable. According to the criteria for the effectiveness of regression, if there is a moderating effect, the OP variable, the MGL, and the interaction term between the two should all hold statistical significance. This prerequisite establishes the determination of whether a moderating effect is present. Column (1) in Table 5 displays the results for the entire sample. The coefficient for OP is −0.081, significant at the 5% level, indicating that organizational politics can diminish work engagement. The MGL holds a positive and significant at the 5% level, suggesting that the doctrine of the mean can enhance employees’ work engagement. The coefficient of the interaction term between organizational politics and the moderate variable is positive and significant at the 1% level. This implies that when organizational politics has a negative impact on work engagement, the presence of the middle-ground ideology can mitigate the detrimental effects brought about by organizational politics. Further, we divided the sample into two groups based on the mean value of MGL: one with higher doctrine of the mean and the other with lower. Then we created a moderation effect graph of the doctrine of the mean, as shown in Figure 1. It can be observed that the moderating effect of higher doctrine of the mean is greater than that of lower. This finding confirms the inference of H3.

**TABLE 5:** Results of moderation effect testing.

	Entire sample		Male		Female	
	(1)		(2)		(3)	
Variables	Coeff	*T*-value	Coeff	*T*-value	Coeff	*T*-value
OP	-0.081**	-2.30	-0.052**	-2.01	-0.034**	-2.17
MGL	0.501***	3.32	0.265***	2.41	0.292***	2.56
OP ( MGL	0.079***	2.51	0.052**	2.06	0.083***	3.66
AGE	0.001	0.08	0.238	0.21	0.301	0.79
EDUCATION	-0.048	-0.55	-1.371*	-1.66	0.761	-0.55
GENDER	0.018	1.59	0.221	0.78	0.237	0.69
Constant	0.031	1.09	1.095	0.97	0.653	1.34
*N*	3059		1987		1072	
R^2^	0.321		0.267		0.350	

*indicates *p* < 0.05,

**indicates *p* < 0.01,

***indicates *p* < 0.001.

Furthermore, we have also tested H4, and the results are presented in column (2) and (3) of Table 5. We found that the coefficient of the interaction term between organizational politics and the moderate variable in column (2) is lower than in column (3), indicating that compared to males, the weakening effect of the doctrine of the mean on the negative impact of organizational politics is relatively greater among females.

We further subgrouped based on age. Column (1) in Table 6 represents the results for employees below 30, where we observed that the coefficient of the interaction term between organizational politics and the doctrine of the mean is not significant, indicating that among individuals aged below 30, the doctrine of the mean is not the primary guiding philosophy for their work behavior. In column (2), the negative and significant impact of organizational politics persists, but the interaction coefficient between organizational politics and the doctrine of the mean is not significant. This suggests that for employees in the age group of 30 to 50, the doctrine of the mean does not diminish the negative impact of organizational politics on their work enthusiasm. The results in column (3) indicate that for employees approaching retirement, the doctrine of the mean can mitigate the negative impact of organizational politics. The aforementioned results indicate the presence of age-related heterogeneity in the moderating effect of the doctrine of the mean on the adverse impact of organizational politics.

**TABLE 6:** Results of moderation effect testing based on age.

	(1)		(2)		(3)	
	Below 30		Aged 30–50		Aged 50 and above	
Variables	Coeff	*T*-value	Coeff	*T*-value	Coeff	*T*-value
OP	-0.177	-1.01	-0.310**	-2.02	-0.419	-1.12
MGL	0.010	1.08	0.052	1.32	0.102***	3.61
OP ( MGL	0.016	0.97	0.012	1.47	0.162***	2.78
AGE	-0.005	-0.76	-0.017	-0.62	0.003	0.74
EDUCATION	-0.038**	-2.44	-0.667	-0.71	0.054	0.67
GENDER	0.032	0.31	-0.015*	-1.88	0.072	0.80
Constant	0.276	0.67	1.677	0.85	-0.271	-0.97
*N*	1043		1144		872	
R^2^	0.203		0.239		0.273	

*indicates *p* < 0.05,

**indicates *p* < 0.01,

***indicates *p* < 0.001.

## 5 Discussion

### 5.1 Comparison with previous studies

Comparison with previous studies, our findings regarding the impact of organizational politics on job involvement are consistent with the research of Tewolde (2023), Lalive et al. (2023), which reinforces the robustness of the conclusions. The above studies underscored how political behaviors in organizations demotivate employees and decrease their commitment and job satisfaction. Our study uniquely contributes to the existing literature by revealing a significant negative relationship between organizational politics and employee work engagement, which is moderated by the principles of the doctrine of the mean. Moreover, our study extends these findings by incorporating the doctrine of the mean. While previous research primarily focused on Western theoretical frameworks, our study adds a unique Eastern perspective. The doctrine of the mean, emphasizing moderation, harmony, and balance, appears to mitigate the negative effects of organizational politics. This aligns with works like Hong et al. (2023), which suggested that traditional Chinese philosophies might provide a buffer against workplace stressors. The applicability of the doctrine of the mean as a moderating factor underscores the importance of considering cultural contexts in organizational studies.

### 5.2 Theoretical implications

This finding resonates with Hofstede’s cultural dimensions theory, which argues that cultural values significantly influence organizational behavior (Taras et al., 2023). However, our study contributes a novel angle by applying a specific Chinese philosophical concept to this dynamic. Our findings also corroborate Triandis’ cultural syndrome theory, which emphasizes the interconnectedness of beliefs, emotions, and behaviors within a culture, forming a syndrome (Triandis, 1996). Within this framework, the doctrine of the mean, as a cultural belief, may intertwine with gender-related behaviors and attitudes, creating a specific cultural syndrome. This could elucidate its varying impacts across different genders. Furthermore, our study expands upon Hall’s cultural communication theory, highlighting the differences in communication styles across cultures, including context and perception of time (Nichol et al., 2023). The role of individuals in communication and decision-making processes may be influenced by cultural values, such as the doctrine of the mean. This may explain how gender differences impact the application of these values. In summary, our findings suggest that the application and influence of cultural values, like the doctrine of the mean, may vary among different genders due to differences in gender-related behavioral norms, communication styles, and belief systems. Our findings contribute to a deeper understanding of these variances and can inform and guide future research in similar domains.

### 5.3 Managerial implications

In terms of practical implications, our findings suggest that management strategies incorporating cultural philosophies like the Doctrine of the Mean could be beneficial. This echoes with the recommendations of Khan et al. (2018), who advocated for management practices aligning with local cultural values. Our study suggests several important implications for management. Firstly, it is essential to minimize organizational political behaviors. To achieve this, university libraries should develop and strengthen a comprehensive institutional regulatory framework. This framework should aim for transparent and accountable processes in critical operations, such as salary distribution and job promotion, thereby reducing the scope for organizational politics. Leaders should exemplify adherence to these regulations and embrace a people-oriented approach that values knowledge and talent, optimizing the use of human resources. Secondly, enhancing the ethical and cultural education of library management personnel is crucial. Regular training sessions focused on traditional culture, especially the doctrine of the mean, can cultivate high moral standards and deter extreme behaviors. Additionally, providing psychological counseling, facilitated by expert psychologists, can help personnel learn self-relaxation techniques, manage anxiety, alleviate work stress, and thus minimize the negative impact of organizational politics on work engagement.

## 6 Conclusion

This study, which analyzed survey data from 3,059 questionnaires completed by university library management personnel, examines the impact of organizational politics on their work engagement. The study yielded the following conclusions: Organizational politics have a significant detrimental effect on the work engagement of library personnel. The impact of organizational politics on work engagement varies with age; it is not significant for individuals below 30 or above 50, but it is notable for those aged between 30 and 50. The doctrine of the mean serves to moderate the negative influence of organizational politics on work engagement. The moderating influence of the doctrine of the mean is stronger among female personnel than their male counterparts.

### 6.1 Limitations

Our study has several limitations that need to be addressed in future research. Firstly, our sample comprises employees from libraries in China’s economically advanced eastern regions. The impact of organizational politics on work engagement in privately operated libraries, as well as in university libraries in the economically less developed central and western regions of China, remains to be further investigated. Secondly, in terms of moderating variables, our focus was on the doctrine of the mean. However, in China, Daoism’s philosophy of inaction, alongside the doctrine of the mean, guides many people’s behaviors. The extent to which daoism’s philosophy of inaction influences the impact of organizational politics on work engagement is currently unclear. Future studies should consider these aspects to more comprehensively elucidate the potential effects of organizational politics on work engagement in the context of cultural diversity.

### 6.2 Future research

The exploration of whether the doctrine of the mean’s moderating effect on organizational politics varies across different jobs as a key future research direction. In China, job positions are primarily categorized into two types: government-funded positions in government agencies, universities, hospitals, and state-owned enterprises, and roles in private or foreign companies. The former, supported by government funding, typically offers employees lower job stress, better benefits, and stability. In contrast, employees in the private and foreign sectors prioritize salary levels due to the absence of government support. Given these divergent work objectives based on job categories, investigating how the doctrine of the mean influences organizational politics differently according to the type of employment presents a fascinating subject for study.

## Data availability statement

The raw data supporting the conclusions of this article will be made available by the authors, without undue reservation.

## Ethics statement

The studies involving humans were approved by the Economic Research Center, affiliated with Shandong University of Finance and Economics. The studies were conducted in accordance with the local legislation and institutional requirements. The participants provided their written informed consent to participate in this study.

## Author contributions

WS: Conceptualization, Investigation, Software, Writing – original draft. CX: Data curation, Formal analysis, Funding acquisition, Methodology, Project administration, Resources, Supervision, Visualization, Writing – original draft.
